# The Acute Effects of Foam Rolling on Fatigue-Related Impairments of Muscular Performance

**DOI:** 10.3390/sports6040112

**Published:** 2018-10-05

**Authors:** Edward Jo, Gabriela A. Juache, Desiree E. Saralegui, Douglas Weng, Shayan Falatoonzadeh

**Affiliations:** Human Performance Research Laboratory, Department of Kinesiology and Health Promotion, California State Polytechnic University Pomona, Pomona, CA 92805, USA; gajuache@cpp.edu (G.A.J.); d.saralegui93@gmail.com (D.E.S.); doug.weng@yahoo.com (D.W.); shayanf11@gmail.com (S.F.)

**Keywords:** foam rolling, vertical jump, ergogenic, skeletal muscle

## Abstract

The purpose of this study was to examine the effects of self-myofascial release (MFR) via foam rolling immediately following strenuous activity on acute fatigue-related impairments of muscular performance. Healthy male (*n* = 16) and female (*n* = 9) subjects visited the laboratory three separate times. During visit 1, subjects were familiarized with performance testing procedures and the foam rolling and fatigue protocols. For visits 2 and 3, subjects were (T1) assessed for vertical jump height, velocity, and power and dynamic reaction time (DRT). Subjects then performed the exercise fatigue protocol, followed by either a foam rolling treatment (MFR) or seated rest (CON). Immediately after, subjects repeated the performance tests (T2). CON resulted in a greater percent decline from T1–T2 for average power (*p* = 0.03), average velocity (*p* = 0.02), and peak power (*p* = 0.03) than the MFR treatment. No between-treatment differences were detected for %∆ vertical jump height (*p* = 0.14) or DRT (*p* = 0.20). According to magnitude-based inference analysis, MFR is likely beneficial in attenuating fatigue-induced kinematic decrements (i.e., power and velocity). Based on magnitude-based inference analysis, MFR is “possibly beneficial” with respect to mitigating acute fatigue-related impairment of jump height and dynamic reaction time. Results demonstrate the plausible short-term benefits of foam rolling on muscular performance decrements associated with acute muscular fatigue from exercise.

## 1. Introduction

Acute performance deficits in athletes during exercise or competition typically result from skeletal muscle fatigue [1]. The underlying mechanisms of exercise-induced fatigue are presently understood as a multifaceted process from peripheral to central factors. From the perspective of integrative physiology, the contractile and force generating machinery of skeletal muscle, known as cross-bridge cycling, is supported by multiple organ and metabolic systems, such as the nervous, cardiovascular, and bioenergetic systems [2]. Failure of any one of these systems to adequately support the contractile or metabolic demands of a given muscle or muscle group would result in diminished force production, muscular work, and thus, overall performance. Therefore, practical strategies to alleviate acute exercise-induced fatigue aim to target these systems to preserve muscular performance capacities during exercise or sport competition. Methods such as nutritional supplementation, ice therapy, compression garments, and active rest have been widely implemented by athletes and exercising individuals with perhaps a disproportionate level of scientific scrutiny.

One of the most widely utilized modalities to facilitate recovery of muscle function and performance following strenuous exercise or activity is self-myofascial release (MFR), an overarching term for self-assisted manual soft tissue therapy. Self-MFR may be performed through various ways, such as foam rolling, which arguably may be the most commonly utilized of the current MFR methods. Foam rolling therapies have largely been adopted by the sport and fitness communities, due to both their practicality and the body of supporting evidence for performance recovery and muscular pain management [3,4,5,6,7]. For instance, massage-like mechanical pressure, as during foam rolling, may potentiate analgesic effects and muscular recovery neurologically by mediating pain-modulatory systems (e.g., nociceptor and mechanoreceptor sensitivity), physiologically via improved circulation, or mechanically through rearrangement of myofascia, muscle fibers, and microvessels [3,8,9,10]. While these effects provide insight as to the potential benefits of MFR to exercise and sport performance, the application of such evidence may be limited in terms of the conditions or situations in which MFR would be most advantageous.

Prior investigations on the acute effects of foam rolling on subsequent performance have yielded mixed results. As presented in a systematic review by Beardsley et al. [11], of the eight studies examining acute performance outcomes [12,13,14,15,16,17,18,19], only one presented evidence indicating improvement (albeit small) on subsequent performance tests (i.e., vertical and long jump, pro-agility, and bench press strength) [17]. The remaining studies showed no acute effect (positive or negative) of foam rolling on performance. It may be concluded that pre-exercise application of foam rolling or similar self-MFR techniques generally fails to provide any immediate ergogenic value. Notably, these investigations have only examined the acute efficacy of self-MFR when applied during resting, non-fatigued situations, begging the question of whether there are other scenarios in which foam rolling may be immediately beneficial for performance.

One of the many settings in which athletes incorporate foam rolling is during situations of exercise-induced muscular fatigue, such as during competition (e.g., half-time break) or a training bout (e.g., intra-workout rest periods). In these circumstances, foam rolling is used with the general intent of acutely mitigating fatigue-induced impairments of performance and preserving functional capacities of the neuromuscular system throughout the remaining period of competition or exercise. However, the efficacy of this strategy remains vaguely understood and is predominately reinforced by anecdotal claims of reduced tightness, improved mobility, and preserved strength. Thus, a scientific investigation would be warranted to better understand this specific self-MFR application. Considering prior yet limited evidence [17] indicating the benefits of pre-exercise foam rolling on unloaded, power-centric movements, such as vertical jump, it would be worthwhile to examine the effects of self-MFR on fatigue-induced declines of similar measures. Therefore, the purpose of this study was to examine the effects of self-MFR via foam rolling immediately following strenuous activity on acute fatigue-related impairments of muscular performance.

## 2. Materials and Methods

### 2.1. Experimental Approach to the Problem

**Figure 1:**
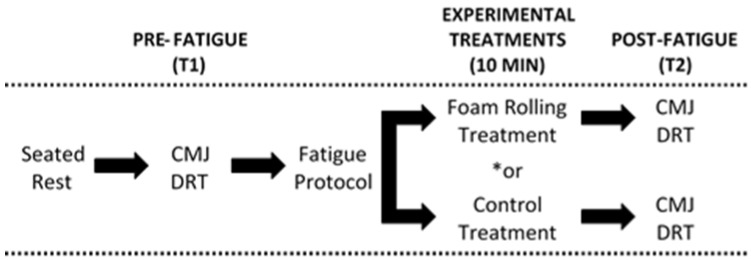
Schematic of Visits 1 and 2. * Experimental treatments were implemented in a crossover manner. CMJ = Countermovement jump w/power and velocity assessment. DRT = Dynamic reaction time.

This was a crossover design study of a convenience sample of Exercise Science university students. Subjects visited the Human Performance Research Laboratory at California State Polytechnic University, Pomona on three separate occasions, each separated by 7 days. During the initial visit, following basic anthropometric measurements, subjects were familiarized with performance testing procedures, which included a countermovement vertical jump test and a dynamic reaction time test. Afterwards, subjects were familiarized with the self-MFR foam rolling protocol as described below. Subjects were then instructed to perform a familiarization bout of the fatigue protocol, also detailed below. For the second and third visits (Figure 1), subjects initially rested in a seated position for 10 min while undergoing pre-exercise blood pressure and heart rate measurements. Afterwards, subjects performed 3 trials of a countermovement vertical jump test while a linear position transducer was used to assess movement velocity and power and a Vertec system (Sports Imports, Columbus, OH, USA) to measure maximum vertical jump height. Then, a dynamic reaction time test was performed using an optical timing system (FITLIGHT Sports Corp., Aurora, ON, Canada). Subjects then performed the exercise fatigue protocol. Subsequently, subjects either underwent a self-MFR foam rolling treatment (MFR) or seated rest (CON). Immediately after, subjects repeated the performance test procedures. Subjects repeated all procedures in the subsequent visit; however, the alternate treatment was implemented, and the order of treatment was counterbalanced. Subjects were asked to maintain normal physical activity/exercise levels during the study time span and to refrain from heavy exercise within 48 h prior to the second and third visits. Dietary intake of the second and third visit was controlled via dietary logs and testing occurred during the same time of day. This study was approved by the Institutional Review Board and was conducted in accordance with the Declaration of Helsinki.

### 2.2. Subjects

Healthy college-aged, recreationally trained male (*n* = 16) and female (*n* = 9) subjects volunteered for this investigation. Subjects met the following inclusion criteria: (1) Age = 18–25 years and (2) recreationally active (i.e., conditioning exercise 2–3 days/week, 30–60 min/day, for the past 6 months prior to the start of the study). Subjects were excluded from participation if they reported or exhibited: (1) A history of medical or surgical events in which the study protocols would be contraindicated or confound the interpretation of results. These included, but were not restricted to, inflammatory, cardiovascular, metabolic, pulmonary, renal, or kidney diseases, hypertension, or musculoskeletal impediments; (2) use of any medication including those with cardiovascular, pulmonary, thyroid, hyperlipidemic, hypoglycemic, hypertensive, endocrinologic, psychotropic, neuromuscular, neurological, androgenic, or anti-inflammatory implications; (3) pregnancy; (4) daily use of ergogenic aids or dietary supplements within 6 weeks prior to the study; or (5) daily use of anti-inflammatory or analgesic agents, for example, non-steroidal anti-inflammatory drugs, pain relievers, muscle relaxers. All subjects signed an informed consent form prior to participation.

### 2.3. Experimental Treatment Protocol

The MFR treatment was self-administered via dynamic foam rolling using a commercially available device composed of a hard inner plastic core enclosed within a layer of ethylene vinyl acetate high density foam (The Grid Foam Roller, Trigger Point Technologies, Austin, TX, USA). The self-MFR treatment was performed bilaterally on the hamstrings, quadriceps, and calves for 30 s each and unilaterally on the hip adductors and the iliotibial tract for 30 s on each side, with slow movements at constant pressures between the origin and insertion of the muscle. A total of 2 bouts were performed, followed by a 3-min seated rest for a total of 10 minutes. This protocol was adapted from a previous study [7]; however, it was extended by one additional bout to better represent longer-duration break periods within athletic competition scenarios. To standardize the foam rolling rate, each stroke had a duration of one second (assisted by metronome) [20]. Subjects were instructed to maintain a subjective pain intensity of seven on a 10 mm visual analogue scale (mild to moderate pain) while applying the treatment. For the control treatment (CON), subjects remained seated for 10 min.

### 2.4. Fatigue Protocol

Muscular fatigue was induced by means of a maximal graded treadmill exercise protocol and a single bout of depth jumps. The treadmill protocol included three 70-s stages of increasing intensity (Stage 1 = 5.5 km/h, 5% incline, Stage 2 = 6.8 km/h, 6% incline, and Stage 3 = 8.0 km/h, 7% incline), followed by a fourth stage at 9.7 km/h and 8% incline until volitional failure, in which the subject has determined he or she is unable to maintain the treadmill speed. Afterwards, subjects performed 3 sets of 10 depth jumps using a 61 cm step-off height. Pilot testing of these procedures demonstrated a significant (*p* < 0.001) 10% decline in vertical jump height, indicating inducement of muscular fatigue.

### 2.5. Vertical Jump Test Procedures

Standing maximum countermovement jump (CMJ) height was measured using a Vertec jump height assessment device (intraclass correlation (ICC) = 0.92, JUMPUSA.com, Sunnyvale, CA, USA). Additionally, during this test, jump kinematics (velocity and power) were measured using a linear position transducer (Tendo Power Analyzer, Tendo Sports Machines, Trencin, Slovak Republic) (ICC = 0.96). The free end of the tether cable was attached to a belt at the posterior center, slightly above the pelvis. Native data acquisition software was utilized to attain and compute dependent variables, which included mean and peak power and velocity. Subjects were provided 4 trials, separated by 1 min of rest. The highest score was subsequently used for analysis.

### 2.6. Dynamic Reaction Time Test Procedures

Dynamic reaction time (DRT) was assessed using the Fit Light system, which incorporates “pods” that are equipped with touch sensors and LED lights. Three of the Fit Light pods were affixed to a wall 1.5 m above the floor, each 1.5 m apart. Subjects began the test standing in front of the middle pod exactly 3 m away in a 2-point stance with feet shoulder-width apart and knees slightly bent. During the test, a single randomized pod would light up and the subjects were instructed to react as quickly as possible by sprinting towards the light and touching the pod. Subjects repeated this step 5 more times, totaling 6 randomized light triggers for a single trial. Subjects performed 3 trials, each separated by 3 min of rest. The average reaction time for each trial was computed and the best score was used for analysis. The DRT time test demonstrated good test–retest reliability (ICC = 0.89).

### 2.7. Data Analysis

The percent delta change (%∆) from baseline (pre-fatigue protocol) (T1) to post-fatigue protocol and treatment (T2) for each outcome measure was computed and used for analysis. A 2 sex by 2 treatment, mixed factor analysis of variance (ANOVA) was used for comparisons across treatments for %∆ average power and velocity, peak power and velocity, jump height, and DRT. A significant mean difference or interaction was determined if the p-value was less than 0.05. Data are presented as mean ± standard deviation, and analysis was performed using SPSS 22 (IBM SPSS Statistics, SPSS Inc., Chicago, IL, USA). Magnitude-Based Inference analysis was implemented as described previously [21,22] to identify clinically meaningful differences between treatments in the %∆ from T1–T2 of each performance measure. The threshold values for positive and negative effects were calculated by multiplying the standard deviations of the control %∆ values by 0.2 (i.e., smallest worthwhile effect) [22]. The probability of the effect was evaluated according to the following scale: <0.5% = most unlikely; 0.5–5% = very unlikely; 5–25% = unlikely; 25–75% = possibly; 75–95% = likely; 95–99.5% = very likely; >99.5% = most likely.

## 3. Results

**Figure 2:**
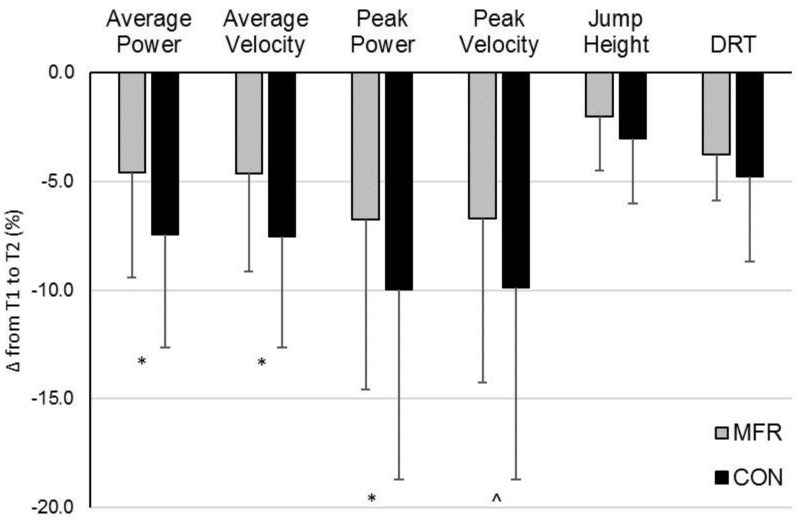
Percent change in performance measures from T1 to T2 between self-myofascial release foam rolling (MFR) and control (CON) treatments. Values presented as mean ± SD. T1 = baseline/pre-fatigue protocol, T2 = post-fatigue protocol + treatment, DRT = Dynamic Reaction Time. * Significantly different between treatments (*p* < 0.05). ^ between-treatment comparison (*p* = 0.06).

The CON treatment resulted in a significantly greater percent decline from T1–T2 for average power, average velocity, and peak power than the MFR treatment (Figure 2) (average power: MFR = −4.6 ± 4.8% vs. CON = −7.4 ± 5.2%, *p* = 0.03, 95% CI = 0.3, 5.4) (average velocity: MFR = −4.7 ± 4.5% vs. CON = −7.6 ± 5.1%, *p* = 0.02, 95% CI = 0.4, 5.4) (peak power: MFR = −6.8 ± 7.8% vs. CON = −10.0 ± 8.7%, *p* = 0.03, 95% CI = 0.3, 6.8). Between-treatment difference in %∆ peak velocity approached significance (*p* = 0.06). No between-treatment differences were detected for %∆ CMJ height (*p* = 0.14) or DRT (*p* = 0.20). According to magnitude-based inference analysis, the MFR is likely beneficial in attenuating kinematic decrements associated with acute exercise-induced fatigue with regards to average and peak power and velocity (Table 1). MFR is possibly beneficial with respect to mitigating acute fatigue-induced impairment of vertical jump height and dynamic reaction time.

## 4. Discussion

The results of the present study demonstrate the plausible short-term benefits of self-MFR via foam rolling on muscular performance decrements associated with acute muscular fatigue from strenuous exercise. However, several key study limitations must be considered within the interpretation and will be discussed below. In sum of our findings, foam rolling following an exercise fatigue protocol blunted the fatigue-induced attenuation of movement velocity and power during a maximum vertical jump test. However, these observed differences in fatigue-related alterations to kinematic variables failed to manifest in any significant between-treatment distinctions in direct performance measures (i.e., vertical jump height and DRT). It also remains unclear how these observed effects on vertical jump kinematics would translate to performance of other sport-specific movements. A wider spectrum of performance tests would be warranted for future investigations to better understand the practical implications of these data to sport and exercise. Qualitative inferences of the effect magnitude for direct performance outcomes were “possibly beneficial”, despite stronger inferences for underlying kinematic measures. Thus, a level of uncertainty remains with respect to the efficacy by which foam rolling blunts performance impairments provoked by strenuous exercise.

Several studies have examined the acute effects of self-MFR techniques (via foam rolling or massage roller) on performance. Although these prior experimental self-MFR treatments were not investigated under acutely fatigued conditions, their findings may provide some insight to the overall effects of self-MFR on performance. Of the few investigations that have shown performance gains following pre-exercise self-MFR, Halperin et al. [16] demonstrated an increase in both range of motion (ROM) and maximum force output of the ankle and calf muscles, respectively, following 3 sets of 30 s of self-MFR treatment via roller massage when compared to control. However, most prior studies failed to show any effect (positive or negative) of pre-exercise self-MFR on subsequent performance measures [12,14,15]. What remains more scientifically corroborated is the positive effect of self-MFR on ROM as opposed to direct performance measures [12,14,23]. For instance, MacDonald et al. [12] showed an 8–10 degree improvement in ROM following an acute bout of foam rolling without a concomitant effect on muscular performance or contractile force capacities. In other studies, improvements in ROM were observed following treatment durations of 1.5 to 3 min [24]. Although the current investigation lacked the appropriate assessments, an improvement in ROM may at least partly explain the preservation of jump kinematics under exercise-induced fatigue. This, of course, is assuming that the decline in performance following the fatigue protocol was, to some degree, associated with reduced ROM. It would be particularly insightful to further examine whether the observed preservation of performance kinematics is related to any influence of self-MFR on fatigue-induced changes to ROM. This presents a limitation to the present study, as a more comprehensive assessment of movement kinematics which includes ROM would provide further information regarding velocity/power changes. Overall, an increase in ROM with no observed impairment of performance may be of practical or clinical value. Altogether, the evidence suggests that self-MFR therapies, when applied pre-exercise, fail to inhibit or improve subsequent performance; however, this conclusion is predicated on a limited pool of studies ranging from fair to high methodological quality, as previously stated in a systematic review [24].

It appears, from the current data, however, that the ergogenic value of self-MFR is not relevant to performance enhancement per se, but rather performance preservation during exercise-induced fatigue. This highlights the novelty of the present study, since prior investigations have only examined the acute effects of self-MFR on performance during non-fatigued, “warm-up” settings. However, as alluded to earlier, current findings must be interpreted with caution, as this study presents data from a limited and relatively heterogenous subject pool. Nonetheless, the results provide further justification for additional research that may strengthen the current evidence supporting the use of self-MFR or foam rolling specifically in acute fatigue scenarios. The current study presents limitations related to the use of seated rest as a control treatment. It is certainly plausible that the observed effect of self-MFR was confounded by a possible psychosomatic interference. In other words, fatigued subjects may have performed better following the self-MFR treatment compared to the control simply because subjects may have the prenotion that foam rolling should improve performance. A pragmatic approach to this limitation would be the inclusion of a sham control treatment. In a previous study by Arroyo-Morales et al. [25], an experimental massage-MFR treatment was investigated in comparison to a sham ultrasound control. This was an elegant control strategy that blinded subjects to the experimental treatment and may have ameliorated any psychological interference of the observed effects. Additionally, an assessment of perceived levels of fatigue may have added valuable insight to the potential interaction between perception of fatigue and performance preservation during fatigued situations.

There appears to be some merit for the use of foam rolling during intra-workout/competition settings for the acute mitigation of fatigue-induced impairment of performance kinematics (i.e., power and velocity). Our data demonstrated the advantages of a 7-min foam rolling treatment compared to passive rest on the preservation of velocity and power following strenuous exercise. However, foam rolling failed to alter fatigue-related decrements to CMJ height and DRT, indicating the possibility that the conservation of underlying kinematic factors like power and velocity was not robust enough to improve direct performance outcomes. At the very least, foam rolling intra-exercise, pre-exercise, or post-exercise does not appear to exert any negative effects on subsequent performance and, therefore, presents very little risk to the athlete. When considering the overall body of research examining the immediate effects of self-MFR on performance, it appears that the benefits of foam rolling may be better observed by the athlete when it is incorporated during intra-competition/workout as opposed to a pre-competition/exercise warm-up routine. Future investigations specifically with respect to our research query should incorporate comparisons of foam rolling against a sham control treatment or other popularized methods of performance preservation under fatigued conditions, such as active rest, dynamic stretching, static stretching, or other methods of self-MFR.

## Figures and Tables

**Table 1:** Percent change in performance variables from T1 to T2 in MFR vs. CON treatments, and qualitative inferences (QI) for the effects of MFR on each variable.

Variable (%∆ from T1–T2)	MFR (Mean ± SD %)	CON (Mean ± SD%)	Mean Difference (Mean ± SD%)	*p*-Value	QI for Effect Magnitude (Mean Difference ± 90% CL)
Avg. Power	−4.6 ± 4.8	−7.4 ± 5.2	2.8 ± 6.2	0.03	Likely beneficial (2.8 ± 2.1)
Avg. Velocity	−4.7 ± 4.5	−7.6 ± 5.1	2.9 ± 6.0	0.02	Likely beneficial (2.9 ± 2.1)
Peak Power	−6.8 ± 7.8	−10.0 ± 8.7	3.6 ± 7.9	0.03	Likely beneficial (3.6 ± 2.7)
Peak Velocity	−6.7 ± 7.5	−9.9 ± 8.8	3.2 ± 8.0	0.06	Likely beneficial (3.2 ± 2.8)
Vertical Jump Height	−2.0 ± 2.5	−3.0 ± 3.0	1.0 ± 3.4	0.15	Possibly beneficial (1.0 ± 1.2)
Dynamic Reaction Time	−3.8 ± 2.1	−4.8 ± 3.9	1.0 ± 3.9	0.20	Possibly beneficial (1.0 ± 1.3)

T1 = baseline/pre-fatigue protocol, T2 = post-fatigue protocol + treatment, MFR = Self-Myofascial Release Treatment, CON = Control Treatment, CL = Confidence Limit.
